# Elevated Lactate Dehydrogenase (LDH) level as an independent risk factor for the severity and mortality of COVID-19

**DOI:** 10.18632/aging.103770

**Published:** 2020-08-14

**Authors:** Chang Li, Jianfang Ye, Qijian Chen, Weihua Hu, Lingling Wang, Yameng Fan, Zhanjin Lu, Jie Chen, Zaishu Chen, Shiyan Chen, Junlu Tong, Wei Xiao, Jin Mei, Hongyun Lu

**Affiliations:** 1 Department of Cardiology, Hubei No.3 People’s Hospital of Jianghan University, Wuhan 430033, Hubei Province, China; 2 Department of Endocrinology and Metabolism, The Fifth Affiliated Hospital of Sun Yat-sen University, Zhuhai 519000, Guangdong Province, China; 3 Guangdong Provincial Key Laboratory of Biomedical Imaging, The Fifth Affiliated Hospital of Sun Yat-sen University, Zhuhai 519000, Guangdong Province, China; 4 Department of Emergency, The Fifth Hospital in Wuhan, Wuhan 430050, Hubei Province, China; 5 Department of Respiratory, The First Hospital of Yangtze University, Jingzhou 434000, Hubei Province, China; 6 School of Health Sciences, Wuhan University, Wuhan 430071, Hubei Province, China; 7 Department of Gastroenterology, The Fifth Affiliated Hospital of Sun Yat-sen University, Zhuhai 519000, Guangdong Province, China; 8 Department of Cardiology, Jiayu People’s Hospital, Jiayu 437200, Hubei Province, China; 9 Central Laboratory, Ningbo First Hospital of Zhejiang University, Ningbo 315010, Zhejiang Province, China; 10 Department of Endocrinology and Metabolism, Zhuhai Hospital Affiliated with Jinan University, Zhuhai People’s Hospital, Zhuhai 519000, Guangdong Province, China

**Keywords:** COVID-19, lactate dehydrogenase, risk factor, severity, mortality

## Abstract

Early identification of severe patients with coronavirus disease 2019 (COVID-19) is very important for individual treatment. We included 203 patients with COVID-19 by propensity score matching in this retrospective, case-control study. The effects of serum lactate dehydrogenase (LDH) at admission on patients with COVID-19 were evaluated. We found that serum LDH levels had a 58.7% sensitivity and 82.0% specificity, based on a best cut-off of 277.00 U/L, for predicting severe COVID-19. And a cut-off of 359.50 U/L of the serum LDH levels resulted in a 93.8% sensitivity, 88.2% specificity for predicting death of COVID-19. Additionally, logistic regression analysis and Cox proportional hazards model respectively indicated that elevated LDH level was an independent risk factor for the severity (HR: 2.73, 95% CI: 1.25-5.97; P=0.012) and mortality (HR: 40.50, 95% CI: 3.65-449.28; P=0.003) of COVID-19. Therefore, elevated LDH level at admission is an independent risk factor for the severity and mortality of COVID-19. LDH can assist in the early evaluating of COVID-19. Clinicians should pay attention to the serum LDH level at admission for patients with COVID-19.

## INTRODUCTION

Since the end of 2019, Wuhan, China, has experienced an outbreak of coronavirus disease 2019 (COVID-19) caused by a novel coronavirus later named severe acute respiratory syndrome coronavirus 2 (SARS-CoV-2) [1]. SARS-CoV-2 is an RNA virus that can be transmitted from person to person, and all people are susceptible to this infection. At present, COVID-19 has progressed into a pandemic and become a major global health concern. It is reported that most cases are nonsevere type with a good prognosis; however, severe cases may deteriorate rapidly to multiple organ damage, impaired immune function and even death [2]. Therefore, early identification of severe COVID-19 is very important for individual or precise management, including antiviral, organ support and intensive care unit (ICU) care, to improve the prognosis.

Lactate dehydrogenase (LDH) is an intracellular enzyme involved in anaerobic glycolysis that catalyzes the oxidation of pyruvate to lactate [3]. Serum LDH is routinely tested in various diseases clinically. It has been reported that elevated serum LDH levels are associated with poor prognosis in various diseases, especially in tumors and inflammation [4–6]. To date, studies have shown that patients with severe COVID-19 have elevated serum LDH levels [7, 8], but no study has specifically evaluated its effect on the severity and mortality of COVID-19. Therefore, this multicenter retrospective, case-control study aimed to explore whether the serum LDH levels at admission can assist in evaluating the severity and mortality of COVID-19.

## RESULTS

### Results of propensity score matching and baseline of patients

Sex, age, hypoproteinemia or anemia, tumor history, chronic kidney disease, stroke history, hyperlipidemia, hypertension, diabetes, coronary heart disease, viral hepatitis, smoking and drinking were included as covariates in the logistic regression model of the propensity score matching. We matched 203 patients (128 nonsevere and 75 severe cases) from among 523 patients (424 nonsevere and 99 severe cases) with laboratory confirmed SARS-Cov-2 infection by propensity score matching. The quality assessment of the propensity score matching is shown in Supplementary Figure 1, and the comparison before and after propensity score matching is shown in Table 1. Overall, the results of propensity score matching were satisfactory. After propensity score matching, the difference in covariables between the nonsevere group and the severe group were controlled within no statistical differences (Table 1).

**Table 1:** Baseline of included patients.

	**Before matching**				**After matching**		
	**Nonsevere (n=424)**	**Severe (n=99)**	**P values**		**Nonsevere (n=128)**	**Severe (n=75)**	**P values**
Female	209(49.3%)	39(39.4%)	0.096		52(40.6%)	31(41.3%)	1.000
Age	51.45±15.08	61.54±13.36	<0.001		57.13±14.55	58.49±13.35	0.508
Hypoproteinemia or anemia	24(5.7%)	25(25.3%)	<0.001		13(10.2%)	13(17.3%)	0.208
Tumor history	8(1.9%)	1(1.0%)	0.861		2(1.6%)	1(1.3%)	1.000
Chronic kidney disease	10(2.4%)	7(7.1%)	0.039		6(4.7%)	3(4.0%)	1.000
Stroke history	8(1.9%)	11(11.1%)	<0.001		3(2.3%)	1(1.3%)	1.000
Hyperlipidemia	48(11.3%)	8(8.1%)	0.448		11(8.6%)	7(9.3%)	1.000
Hypertension	82(19.3%)	43(43.4%)	<0.001		44(34.4%)	25(33.3%)	1.000
Diabetes	61(14.4%)	23(23.2%)	0.045		32(25.0%)	18(24.0%)	1.000
Coronary heart disease	17(4.0%)	12(12.1%)	0.003		10(7.8%)	6(8.0%)	1.000
Viral hepatitis	7(1.7%)	1(1.0%)	0.99		3(2.3%)	1(1.3%)	1.000
Smoking	27(6.4%)	13(13.1%)	0.008		10(7.8%)	9(12.0%)	0.566
Drinking	28(6.6%)	16(16.2%)	0.002		12(9.4%)	10(13.3%)	0.628
Death	0	26(26.3%)	<0.001		0	16(21.1%)	<0.001

In the current study, 26 (5.0%) out of 523 patients before propensity score matching and 16 (7.9%) out of 203 patients after propensity score matching died of COVID-19. Considering that the patients were not continuously enrolled, we cannot calculate the case fatality rate.

### Comparison of laboratory indicators between the nonsevere group and the severe group

**Figure 1:**
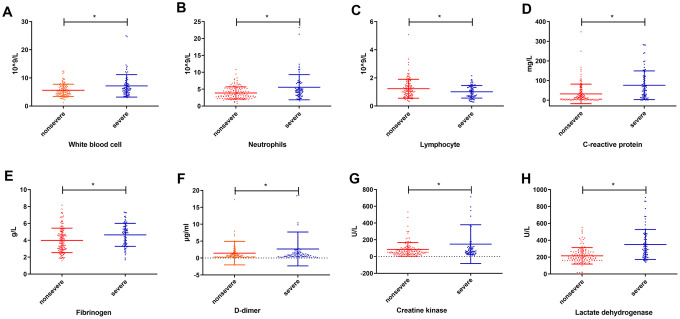
**Levels (mean ± SD) of laboratory indicators at admission between the nonsevere group and severe group.** (**A**) white blood cell; (**B**) neutrophils; (**C**) lymphocyte; (**D**) c-reactive protein; (**E**) fibrinogen; (**F**) d-dimer; (**G**) creatine kinase; (**H**) lactate dehydrogenase. * P<0.05.

We analyzed the levels of laboratory indicators at admission between nonsevere group and severe group. There were significant differences (P<0.05) in the levels of white blood cells (WBCs), neutrophils, lymphocytes, C-reactive protein (CRP), fibrinogen, D-dimer, creatine kinase and LDH between two groups (Figure 1 and Supplementary Table 1). Considering the relationship among laboratory indicators, we conducted Pearson correlation analysis on these laboratory indicators with significant differences. As a result, CRP and LDH exhibited powerful correlations with other indexes (Supplementary Table 2), which suggested that CRP and LDH were significant factors associated with the severity of COVID-19.

### Role of the serum LDH in severity and death among COVID-19 cases

**Figure 2:**
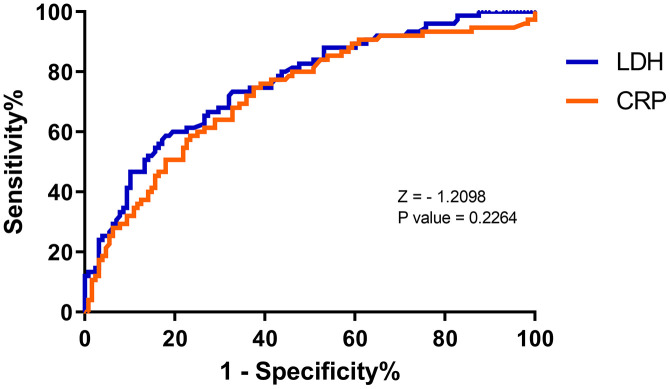
**Receiver operating characteristic (ROC) curve for predicting severity of COVID by C-reactive protein (CRP) and lactate dehydrogenase (LDH) levels at admission.** LDH: AUC 0.76 ± 0.04, cut-off 277.00 U/L, sensitivity 58.7%, specificity 82.0%. CRP: AUC 0.73 ± 0.04, cut-off 20.14 mg/L, sensitivity 74.7%, specificity 62.5%.

We performed ROC curves on the above laboratory indicators with significant differences to assess their value in patients with COVID-19. Lymphocyte counts were the most specific predictor (specificity 94.7%) for severe COVID-19, but with a low sensitivity of 20.3% (Table 2). In contrast, D-dimer had a high sensitivity (86.7%) but a very poor specificity (37.5%) in predicting severe COVID-19. Overall, serum LDH levels had an AUC of 0.76 (95% CI: 0.70 - 0.83) for predicting severe COVID-19, with a 58.7% sensitivity and 82.0% specificity, based on a best cut-off of 277.00 (U/L) (Table 2). However, there seems to be no significant difference between CRP and LDH in predicting severe COVID-19 (Figure 2).

**Table 2:** Role of laboratory indicators in predicting the severity and death of COVID-19.

	**Predicting severity of COVID-19**					**Predicting death of COVID-19**			
	**AUC**	Best cut-off ^*^	**Sensitivity**	**Specificity**		**AUC**	Best cut-off ^*^	**Sensitivity**	**Specificity**
WBC	0.63±0.04	5.65 (×10^9^/L)	0.627	0.594		0.78±0.07	7.45(×10^9^/L)	0.688	0.797
Neutrophils	0.66±0.04	3.85 (×10^9^/L)	0.707	0.586		0.82±0.05	4.87(×10^9^/L)	0.813	0.711
Lymphocyte	0.58±0.04	1.72 (×10^9^/L)	0.203	0.947		0.76±0.06	0.73(×10^9^/L)	0.759	0.750
NLR	0.68±0.04	3.83	0.640	0.660		0.87±0.06	7.42	0.750	0.900
CRP	0.73±0.04	20.14 (mg/L)	0.747	0.625		0.89±0.05	91.39 (mg/L)	0.813	0.882
Fibrinogen	0.64±0.04	4.79 (g/L)	0.533	0.758		0.69±0.06	3.96 (g/L)	0.875	0.497
D-dimer	0.65±0.04	0.33 (μg/ml)	0.867	0.375		0.80±0.06	1.09 (μg/ml)	0.813	0.706
CK	0.55±0.04	109.50 (U/L)	0.347	0.812		0.62±0.08	120.50 (U/L)	0.438	0.818
LDH	0.76±0.04	277.00 (U/L)	0.587	0.820		0.92±0.05	359.50 (U/L)	0.938	0.882

^*^ Chosen by maximizing the Youden index. Abbreviations: AUC, area under the curve; WBC, white blood cell; NLR, neutrophil-to-lymphocyte ratio; CRP, c-reactive protein; CK, creatine kinase; LDH, lactic dehydrogenase.

**Figure 3:**
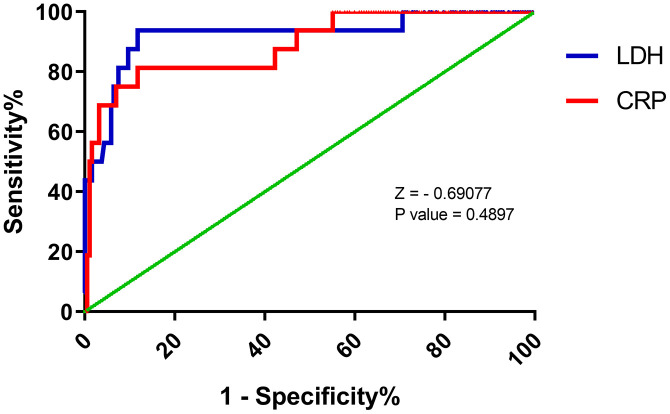
**Receiver operating characteristic (ROC) curve for predicting death (B) of COVID by C-reactive protein (CRP) and lactate dehydrogenase (LDH) levels at admission.** LDH: AUC 0.92 ± 0.05, cut-off 359.50 U/L, sensitivity 93.8%, specificity 88.2%. CRP: AUC 0.89 ± 0.05, cut-off 91.39 mg/L, sensitivity 81.3%, specificity 88.2%.

The AUC values of the above indicators, even the CRP and LDH, were not very satisfactory. Therefore, we further analyzed the role of these indicators in predicting the mortality due to COVID-19. Unexpectedly, a cut-off of 91.39 mg/L for serum CRP levels had a sensitivity of 81.3% and a specificity of 88.2% for predicting death in patients with COVID-19 (Table 2). In addition, when the best cut-off of was 359.50 U/L, serum LDH levels had an AUC of 0.92 (95% CI: 0.84 - 0.99) for predicting death due to COVID-19, with a sensitivity of 93.8% and specificity of 88.2% (Table 2). Similarly, there was no significant difference in the ROC curve between CRP and LDH (Figure 3).

### Elevated serum LDH as an independent risk factor for the severity of COVID-19

We detected the risk factors for the severity of COVID-19 by univariate and multivariate logistic regression analysis. Neutrophils were excluded from logical regression analysis because neutrophils and leukocytes were collinear. In univariate analysis, high levels of WBC (HR: 2.32, 95% CI: 1.29, 4.16; P=0.005), CRP (HR: 4.91, 95% CI: 2.61-9.24; P<0.001), neutrophil-to-lymphocyte ratio (HR: 3.51, 95% CI: 1.93-6.39; P<0.001), fibrinogen, D-dimer (HR: 3.26, 95% CI: 1.60-6.64; P=0.001), creatine kinase and LDH (HR: 6.48, 95% CI: 3.40-12.34; P<0.001), and low levels of lymphocytes (HR: 4.53, 95% CI: 1.51-13.53; P=0.007) were risk factors for the severity of COVID-19 (Table 3). Furthermore, we took indicators that were P<0.1 in univariate logistic regression into multivariate logistic regression analysis. Of the 8 indicators, the P value of the serum LDH levels was still less than 0.05, which suggested that elevated serum LDH (HR: 2.73, 95% CI: 1.25-5.97; P=0.012) is an independent risk factor for the severity of COVID-19 (Table 3).

**Table 3:** Uni- and multivariate logistic regression analyses of risk factors for the severity of COVID-19.

**Variables**	**Univariate logistic regression**			**Multivariate logistic regression**	
	**P value**	**Hazard ratio (95%CI)**		**P value**	**Hazard ratio (95%CI)**
WBC^*^ (> 5.65×10^9^/L)	0.005	2.32 (1.29, 4.16)		0.056	2.01 (0.98, 4.09)
Lymphocyte^*^ (< 1.72×10^9^/L)	0.007	4.53(1.51, 13.53)		0.240	2.09 (0.61, 7.15)
NLR^*^ (>3.83)	< 0.001	3.51 (1.93, 6.39)		0.633	1.21 (0.55, 2.64)
CRP^*^ (> 20.14 mg/L)	< 0.001	4.91(2.61, 9.24)		0.109	1.93 (0.86, 4.31)
Fibrinogen^*^ (> 4.79 g/L)	< 0.001	3.58(1.95, 6.57)		0.257	1.54 (0.73, 3.22)
D-dimer^*^ (> 0.33 μg/ml)	0.001	3.26(1.60, 6.64)		0.398	1.43 (0.62, 3.29)
CK^*^ (> 109.50 U/L)	0.012	2.30(1.20, 4.41)		0.364	1.43 (0.66, 3.08)
LDH^*^ (> 277.00 U/L)	< 0.001	6.48(3.40, 12.34)		0.012	2.73(1.25, 5.97)

^*^Take the best cut-off for predicting the severity of COVID-19 as the boundary value of binary variable. Abbreviations: WBC, white blood cell; NLR, neutrophil-to-lymphocyte ratio; CRP, C-reactive protein; CK, creatine kinase; LDH, lactic dehydrogenase.

### Elevated serum LDH as an independent risk factor for mortality of COVID-19

We applied the Cox proportional hazards model to evaluate the effect of LDH on the survival time of patients. In univariable Cox regression analysis, male sex (HR: 3.04, 95%: CI 0.87-10.65; P=0.083) and age older than 60 years (HR: 5.88, 95% CI: 1.33-25.90, P=0.019) had a significant effect on the survival time of patients. In addition, elevated serum WBC count (HR: 8.06, 95% CI: 2.8-23.23; P<0.001), neutrophil-to-lymphocyte ratio (HR: 21.11, 95% CI: 6.80-65.51; P<0.001), CRP (HR: 24.06, 95% CI: 6.85-84.50; P<0.001), fibrinogen, D-dimer, CK, LDH (HR: 77.20, 95% CI: 10.20-584.61; P<0.001) and reduced lymphocyte counts were risk factors of mortality (Table 4). We take indicators that were P<0.1 in univariate logistic regression into multivariate logistic regression analysis. We found that the elevated serum LDH (HR: 40.50, 95% CI: 3.35-449.28; P=0.003) remained an independent risk factor for the mortality of COVID-19 (Table 4).

**Table 4:** Uni- and multivariate Cox regression analyses of risk factors for the death due to COVID-19.

**Variables**	**Univariate Cox regression**			**Multivariate Cox regression**	
	**P value**	**Hazard ratio (95%CI)**		**P value**	**Hazard ratio (95%CI)**
Sex (male)	0.083	3.04 (0.87, 10.65)		0.876	1.13 (0.25, 5.14)
Age (> 60)	0.019	5.88 (1.33, 25.90)		0.914	1.12 (0.15, 8.13)
WBC^*^ (> 7.45×10^9^/L)	< 0.001	8.06 (2.80, 23.23)		0.245	2.46 (0.54, 11.19)
Lymphocyte^*^ (< 0.73×10^9^/L)	< 0.001	7.47 (2.41, 23.18)		0.843	1.17 (0.24, 5.71)
NLR^*^ (>7.42)	< 0.001	21.11 (6.80, 65.51)		0.131	4.33 (0.65, 28.95)
CRP^*^ (> 91.39 mg/L)	< 0.001	24.06 (6.85, 84.50)		0.558	1.82 (0.25, 13.52)
Fibrinogen^*^ (> 3.96 g/L)	0.016	6.19 (1.41, 27.21)		0.846	1.23 (0.15, 9.76)
D-dimer^*^ (> 1.09 μg/ml)	0.001	8.67 (2.47, 30.45)		0.476	0.51 (0.08, 3.22)
CK^*^ (> 120.50 U/L)	0.023	3.14 (1.17, 8.42)		0.827	1.13 (0.37, 3.41)
LDH^*^ (> 359.50 U/L)	< 0.001	77.20 (10.20, 584.61)		0.003	40.50(3.65, 449.28)

^*^Take the best cut-off for predicting death due to COVID-19 as the boundary value of binary variable. Abbreviations: WBC, white blood cell; NLR, neutrophil-to-lymphocyte ratio; CRP, C-reactive protein; CK, creatine kinase; LDH, lactic dehydrogenase.

## DISCUSSION

In this study, we identified that elevated serum LDH level was an independent indicator for predicting severity and mortality in patients with COVID-19 for the first time. Based on ROC analysis, serum LDH levels at admission had high specificity for predicting the severity of COVID-19 and a satisfactory sensitivity and specificity for predicting death due to COVID-19. Furthermore, logistic regression analysis and Cox proportional hazards model revealed that elevated serum LDH at admission to be an independent risk factor for the severity and mortality of COVID-19.

We regarded sex, age, hypoproteinemia or anemia, tumor history, chronic kidney disease, stroke history, hyperlipidemia, hypertension, diabetes, coronary heart disease, viral hepatitis, smoking and drinking as covariates in the logistic regression model of the propensity score matching, because these covariates may have an impact on the severity and mortality of COVID-19 [9–11]. Autoimmune and inflammatory diseases do have an impact on the severity and mortality of COVID-19. We did not include autoimmune and inflammatory diseases in the logistic regression model of the propensity score matching because there were no patients diagnosed with autoimmune and inflammatory diseases in the enrolled patients. After propensity score matching, the differences in covariables between the nonsevere group and the severe group were controlled at almost the same levels. Controls for confounding factors were the premise of this study, ensuring the reliability of the conclusions.

As suggested by comparison of laboratory indicators, there were significant differences in the levels of WBC, neutrophils, lymphocytes, CRP, fibrinogen, D-dimer, creatine kinase and LDH between nonsevere and severe groups. The differences in these indicators were very similar to those reported by Huang et al. [12]. Notably, LDH showed a powerful correlation with the other indexes by Pearson correlation analysis, which suggested that LDH was a significant factor associated with the severity of patients with COVID-19. When the body experiences acute hypoxia or inflammation, the level of LDH in serum will rise significantly. COVID-19, caused by SARS-Cov-2 infection, mainly involves in the lungs, as well as other tissues and organs [13, 14], leading to hypoxia, thrombogenesis, inflammation and organ injury. Theoretically, elevated serum LDH is an important laboratory indicator for evaluating COVID-19 [15].

In this study, male sex and age older than 60 years old had obvious effects on death due to COVID-19. We found that patients who were aged over 60 years (HR: 5.88, 95% CI: 1.33-25.90, P=0.019) and male (HR: 3.04, 95%: CI 0.87-10.65; P=0.083) were more likely to expire, as suggested by the univariate Cox proportional hazards model. This obtained similar general conclusions as previous studies [16, 17]. However, the effect of age and sex on death due to COVID-19 was reduced in multivariate Cox regression because the risk of age and sex was adjusted for other factors.

Elevated serum LDH as an independent risk factor for COVID-19 is the main conclusion of this study. In univariate analysis, high WBC, NLR, CRP, fibrinogen, D-dimer, creatine kinase and LDH, and low lymphocyte were not only risk factors for severity but also risk indicators for death among patients with COVID-19 (Table 3 and Table 4). Additionally, in multivariate analysis, elevated serum LDH remained an independent risk factor for COVID-19 severity and mortality. A previous study [17], which did not mention the influence of LDH on COVID-19, proved that NLR is an independent risk factor for in-hospital mortality in COVID-19. Therefore, we included the NLR in Cox proportional hazards model. However, in our study, we proved that LDH was a more independent risk factor compared with NLR as suggested by multivariate Cox regression (Table 4).

There are some limitations in this study that should be noted. Firstly, the number of subjects included is to some extent small which limits the statistical power of this study. Nonetheless, the sample size of this study was sufficient to draw our conclusion. Secondly, on a whole, 16 out of 203 patients died of COVID-19 in this study. Considering the small number of deaths, we performed Cox regression instead of logistic regression to analyze the effect of LDH on COVID-19 mortality. Although the 95% confidence interval of HR is slightly lager, it is enough to ensure that elevated serum LDH is an independent risk indicator for death due to COVID-19. Thirdly, although we have controlled the bias by propensity score matching, multiple potential confounders might not have been fully considered. A small number of patients have taken antiviral drugs, antihypertensive drugs, and antidiabetic drugs prior to admission, the effect of past medical history on the results were not studied.

In conclusion, this study revealed that serum LDH at admission was useful in evaluating the disease severity and in-hospital mortality among patients with COVID-19. Further studies are needed to confirm our findings.

## MATERIALS AND METHODS

### Study design and participants

We collected data for 523 adult patients admitted to the hospital with laboratory confirmed SARS-Cov-2 infection in 4 designated tertiary hospitals in Hubei Province, including 2 in Wuhan city and 2 in cities outside Wuhan, Hubei Province, from January 22, 2020 to March 14, 2020. We divided all these 523 patients into two groups: a severe group (severe type and critical severe type of COVID-19) and a nonsevere group (mild type and moderate type of COVID-19).

Considering that this study is a retrospective study, we used propensity score matching [18] to reduce biases and confounders. Ultimately, 203 patients with COVID-19 (75 patients in the severe group and 128 patients in the nonsevere group) were included.

### Inclusion and exclusion criteria

Patients who met all the following criteria were included: (1) ≥18 years old, male or female; (2) laboratory confirmed SARS-Cov-2 infection; (3) complete clinical, laboratory, imaging and outcome data. Patients younger than 18 years old, with uncomplete clinical information because of transferring to other designated hospitals were excluded.

### Ethical considerations

This study was approved by the Ethics Committee of the Fifth Affiliated Hospital of Sun Yat-sen University (ZDWY2020-K173-1). Written informed consent was waived by the Ethics Committee in consideration of the designated hospital for emerging infectious disease.

### Data collection

The data included basic clinical information, diagnosis, comorbidity, and laboratory data at admission including routine blood examination, liver and renal function, myocardial enzyme, blood coagulation, procalcitonin (PCT), CRP and LDH. Additionally, neutrophil-to-lymphocyte ratio (NLR) was calculate. All these data were obtained with a standardized data collection form created by EpiData software (version 3.1). All data were checked by two physicians (Lingling Wang and Jianfang Ye) and a third researcher (Yameng Fan) adjudicated any difference in interpretation between the two primary reviewers.

### Diagnosis and classification of COVID-19

COVID-19 was diagnosed and classified according to the newest “Guidelines for the Diagnosis and Treatment of COVID-19 (Trial Version 7)” [19] by the National Health Commission in China (http://www.nhc.gov.cn/). Clinical condition classification criteria are as follows: (1) mild type - clinical symptoms were mild, and no radiological changes; (2) moderate type - fever, respiratory tract or other symptoms, and pneumonia can be seen on imaging; (3) severe type - respiratory rate ≥ 30 times per minute, or the oxygen saturation is lower than 93% at rest state, or the ratio of arterial partial pressure of oxygen (PaO_2_) and fraction of inspired oxygen (FiO_2_) is lower than 300 mmHg (altitude below 1000 meters), or pulmonary imaging indicate that lung damage deteriorates rapidly within 24 to 48 hours; (4) critical severe type - respiratory failure requiring mechanical ventilation, or signs indicating shock, or multiple organ failure requiring admission to the intensive care unit.

### Statistical analysis

Propensity score matching was performed using open source R software (version 3.6.3, Vienna, Austria) based on the “MatchIt” package [20]. The calipers value was set to 0.03, the matching ratio was 1:2, and the matching method was “nearest”. Statistical analysis was performed using IBM SPSS software (version 25.0, Chicago, USA). Statistical charts were generated using GraphPad Prism software (version 8.0, San Diego, USA). The statistical results are presented as mean ± standard deviation. Continuous data were analyzed by the Student’s t-tests, and the Levene test was used to decide homogeneity of variance. The receiver operating characteristic (ROC) curve, sensitivity, specificity and area under the curve (AUC) were measured to evaluate the levels of laboratory indicators in predicting the severity and mortality of COVID-19. Differences between AUCs were detected by the Z-test. All indicators were further tested by univariate and multivariate logistic regression or Cox regression analysis. The hazard ratio (HR) and 95% confidence intervals (95% CI) are shown. P value less than 0.05 was considered statistically significant.

## Supplementary Material

Supplementary Figure 1

Supplementary Tables
