# Supplementary material for: Elevated Lactate Dehydrogenase (LDH) level as an independent risk factor for the severity and mortality of COVID-19
Source: Aging (Albany NY). 2020 Aug 14;12(15):15670–81. doi: 10.18632/aging.103770 (PMC7467395; doi:10.18632/aging.103770)
Supplement: Supplementary Figure 1 [file aging-12-103770-s001..pdf]

SUPPLEMENTARY FIGURE

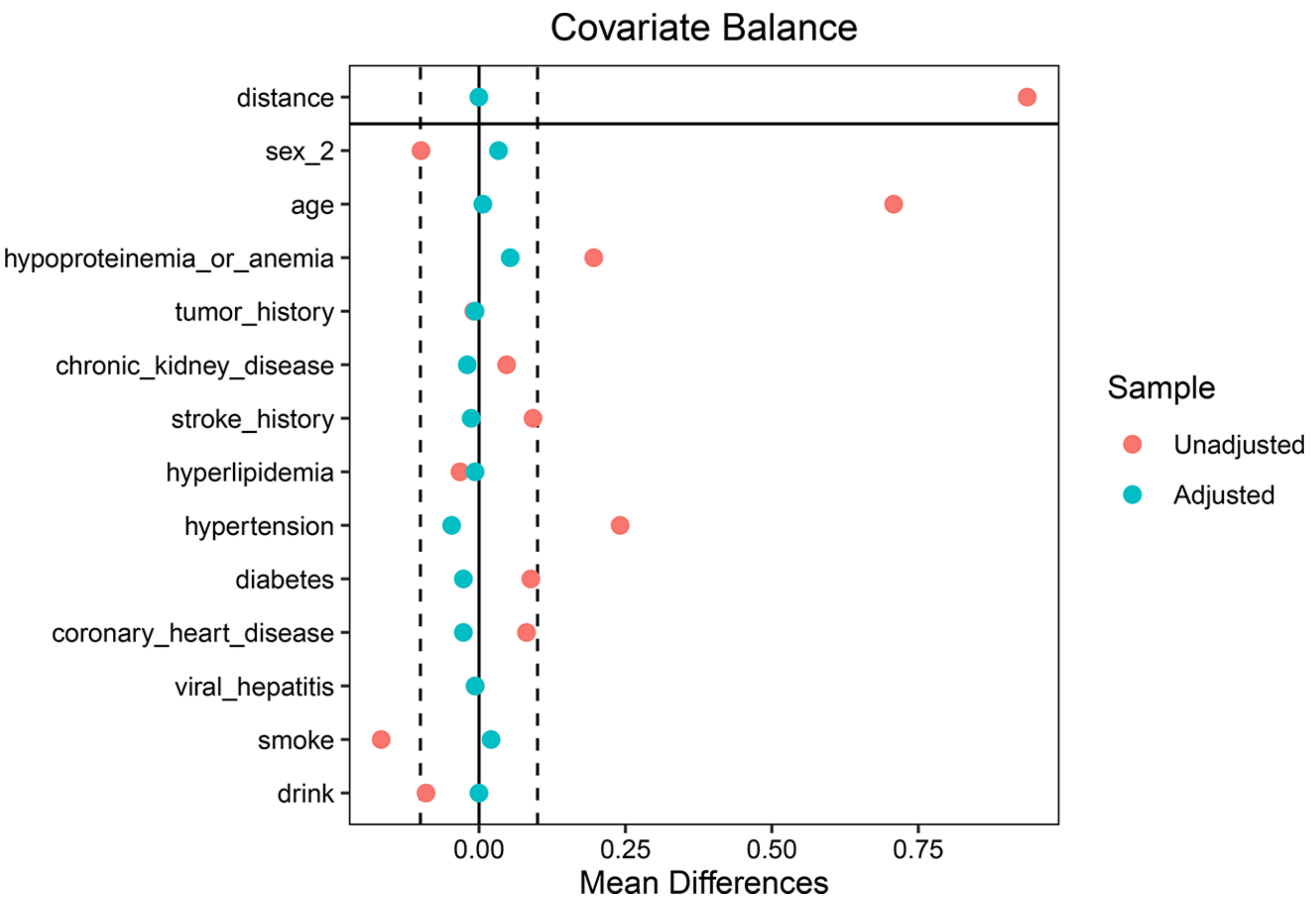

Supplementary Figure 1. Mean differences in covariate balance before and after being adjusted.
