# Supplementary material for: Elevated Lactate Dehydrogenase (LDH) level as an independent risk factor for the severity and mortality of COVID-19
Source: Aging (Albany NY). 2020 Aug 14;12(15):15670–81. doi: 10.18632/aging.103770 (PMC7467395; doi:10.18632/aging.103770)
Supplement: Supplementary Tables [file aging-12-103770-s002..pdf]

SUPPLEMENTARY TABLES

Supplementary Table 1. Laboratory indicators at admission between the nonsevere group and severe group.

	Nonsevere (n=128)	Severe (n=75)	P value*
WBC ($\times 10^9$/L)	5.61±2.16	7.17±3.99	0.002
Neutrophils ($\times 10^9$/L)	3.87±1.81	5.57±3.73	<0.001
Lymphocyte ($\times 10^9$/L)	1.23±0.67	1.01±0.45	0.014
NLR	3.93±3.17	7.2±6.41	<0.001
RBC ($\times 10^{12}$/L)	4.28±0.57	4.41±0.56	0.113
Platelet($\times 10^9$/L)	224.34±103.38	214.08±83.01	0.465
Albumin (g/L)	37.47±5.77	36.12±6.04	0.115
TBIL (μmol/L)	12.38±7.58	13.49±6.89	0.250
DBIL (μmol/L)	4.42±5.63	5.02±3.21	0.401
ALT (U/L)	35.49±32.48	35.61±29.96	0.980
AST (U/L)	33.54±22.04	37.60±22.39	0.209
Creatinine(μmol/L)	83.53±127.53	100.44±150.76	0.395
TG (mmol/L)	1.47±1.11	1.43±0.69	0.814
TC (mmol/L)	4.00±0.99	3.83±0.99	0.261
UA (μmol/L)	272.97±104.19	280.56±113.09	0.628
PCT (ng/mL)	0.20±0.70	0.32±0.90	0.296
CRP (mg/L)	31.84±49.83	75.52±73.09	<0.001
Fibrinogen (g/L)	3.99±1.45	4.65±1.36	0.002
D-dimer (μg/ml)	1.45±3.50	2.69±5.01	0.041
CK (U/L)	85.37±80.53	148.48±231.03	0.025
LDH (U/L)	215.23±97.36	349.28±177.60	<0.001

*Data were analyzed by Student's t-tests and Levene test was used to evaluate homogeneity of variance. Abbreviations: WBC, white blood cell; NLR, neutrophil-to-lymphocyte ratio; RBC, red blood cell; TBIL, total bilirubin; DBIL, direct bilirubin; ALT, alanine aminotransferase; AST, aspartate aminotransferase; TG, triglyceride; TC, total cholesterol; UA, uric acid; PCT, procalcitonin; CRP, c-reactive protein; CK, creatine kinase; LDH, lactic dehydrogenase.

Supplementary Table 2. Pearson correlation coefficient among levels of laboratory indicators.

	WBC	Neutrophils	Lymphocyte	CRP	fibrinogen	D-dimer	CK	LDH
WBC	1.00							
Neutrophils	0.96**	1.00						
Lymphocyte	0.22**	- 0.01	1.00					
CRP	0.31**	0.37**	- 0.37**	1.00				
Fibrinogen	0.13	0.21**	- 0.40**	0.54**	1.00			
D-dimer	0.17*	0.23**	- 0.20**	0.29**	0.04	1.00		
CK	0.02	0.00	- 0.08	0.20**	0.05	- 0.07	1.00	
LDH	0.34**	0.41**	- 0.36**	0.63**	0.34**	0.33**	0.40**	1

*There was a statistical difference at the level of $P < 0.05$. ** There was a statistical difference at the level of $P < 0.01$.
Abbreviations: WBC, white blood cell; CRP, C-reactive protein; CK, creatine kinase; LDH, lactic dehydrogenase.
